# Integrated Application of Transcriptomics and Metabolomics Provides Insights into the Different Body-Size Growth in Chinese Mitten Crab (*Eriocheir sinensis*)

**DOI:** 10.3390/ijms26104617

**Published:** 2025-05-12

**Authors:** Silu Che, Jiancao Gao, Haojun Zhu, Jinliang Du, Liping Cao, Yao Zheng, Gangchun Xu, Bo Liu

**Affiliations:** 1 Wuxi Fisheries College, Nanjing Agricultural University, Wuxi 214081, China; cslcg123456@126.com (S.C.); gaojiancao@ffrc.cn (J.G.); dujl@ffrc.cn (J.D.); caolp@ffrc.cn (L.C.); zhengyao@ffrc.cn (Y.Z.); 2 Key Laboratory of Integrated Rice-Fish Farming Ecology, Ministry of Agriculture and Rural Affairs, Freshwater Fisheries Research Center, Chinese Academy of Fishery Sciences, Wuxi 214081, China; zhuhaojun@ffrc.cn

**Keywords:** Chinese mitten crab (*Eriocheir sinensis*), body-size, transcriptomics, metabolomics

## Abstract

The Chinese mitten crab, *Eriocheir sinensis*, is a water-dwelling crustacean that is widely distributed in northern hemisphere water systems. Body size is one of the crucial indicators determining the economic value of *E. sinensis*. However, research on the genetic basis and regulatory mechanisms of body size in this species is limited, with only a few relevant genes reported. Therefore, it is imperative to investigate the regulatory pathways associated with its growth. This study first utilized transcriptomic profiling and metabolomic sequencing to construct gene expression profiles and metabolite profiles of *E. sinensis* of different body sizes. Subsequently, through integrated omics analysis, the key genes and regulatory pathways involved in controlling the growth and size of crabs were preliminarily identified. This study found that larger female crabs exhibited significantly enhanced digestive functions, primarily reflected in the upregulation of *trypsin-1* expression, suggesting its potentially pivotal role in regulating the growth and development of crabs. Interestingly, a variety of tissue-specific proteins such as APOLPP, RICK A, AGMO, and NEPHRIN, as well as REXO1L1P and ZCCHC24, indirectly influence the growth and development of crabs through their respective functional pathways. In addition, the key KEGG pathways, such as ECM–receptor interaction, cell adhesion, and the PI3K-Akt signaling pathway, were revealed to play central roles in the growth regulation of *E. sinensis*. These findings expand our understanding of the growth regulation mechanisms in crustaceans and offer potential molecular targets for body-size improvement in aquaculture.

## 1. Introduction

The Chinese mitten crab *Eriocheir sinensis* is a migratory species that is widely distributed in northern hemisphere water systems [1]. As a significant aquatic economic species in China, the crab industry holds a prominent position in terms of farming scale, market demand, and cultural value [2,3]. The production of *E. sinensis* reached 888,629 tons in 2023, according to the China Fishery Statistical Yearbook [4]. *E. sinensis*, renowned for its delicious meat and rich nutritional value, enjoys a vast market prospect and is deeply cherished by consumers [5]. The body size of a crab is one of the pivotal economic traits directly influencing market value and consumer preference. Therefore, the body size of *E. sinensis* is a key focus trait for researchers in genetic breeding.

In aquatic vertebrates, body size regulation has been well-explored, and the GH-IGF axis is crucial for regulating body size. In striped bass (*Morone saxatilis*), insulin-like growth factor 1 (IGF-1) regulates the growth by modulating the synthesis and release of growth hormone [6,7]. Similarly, IGF-II plays a critical role in the growth of channel catfish (*Ictalurus punctatus*) [8]. The GH/IGF-1 axis signaling pathway is closely related to the growth regulation of Nile tilapia (*Oreochromis niloticus*) [9]. In addition, molecular markers associated with growth traits have been developed in various fish species, e.g., rainbow trout (*Oncorhynchus mykiss*), mandarin fish (*Siniperca chuatsi*), zebrafish (*Danio rerio*), and turbot (*Scophthalmus maximus*) [10,11,12,13].

In aquatic invertebrates, however, body size regulation has lagged behind relatively. Similar to the study in fish, the IGF-1/AKT signaling pathway influences muscle tissue deposition and growth, thereby regulating the body size of the Pacific oyster (*Crassostrea gigas)* [14]. Meanwhile, insulin-like peptide receptors (ILPRs), such as PI3K-AKT, RAS-MAPK, and TOR, play a significant role in regulating the growth of *C. gigas* [15]. However, research in this field on crustaceans remains relatively scarce, and the molecular mechanisms underlying their size regulation are still not well understood. Currently, research on the growth of Chinese mitten crabs primarily focuses on the genes associated with the molting process. Studies have found that genes such as *cyp15a1*, *cyp3a4*, *EcR*, *MIH*, and chitinase (*chi*) play crucial roles in the molting process of these crabs [16,17,18]. Additionally, phenoloxidase (PO) regulates the growth of these crabs by promoting the hardening of the new exoskeleton after molting [19,20]. Given that only a few genes associated with the growth of *E. sinensis* were reported, further in-depth exploration is urgently needed.

Aiming to explore the growth regulation mechanisms in *E. sinensis*, this study collected crabs of different body sizes from the same pond. By using transcriptomic and metabolomic integrative analysis, we identified differentially expressed genes and pathways associated with the growth phenotypes. These findings will enhance public understanding of the growth regulation mechanisms in crustaceans and provide a scientific basis for genetic selection and breeding improvement in crabs.

## 2. Results

### 2.1. Changes in Gene Expression of Different Body Sizes of E. sinensis

A total of 24 cDNA libraries of muscle tissues of *E. sinensis* were constructed and sequenced. A total of 166.60 Gb of clean data was obtained, with more than 6.94 Gb of each sample. The Q30 ratio was larger than 90.08% for all samples. All clean reads of each sample were compared with the assembled reference genome. The average mapping rate was 88.38% (Table S1).

**Figure 1:**
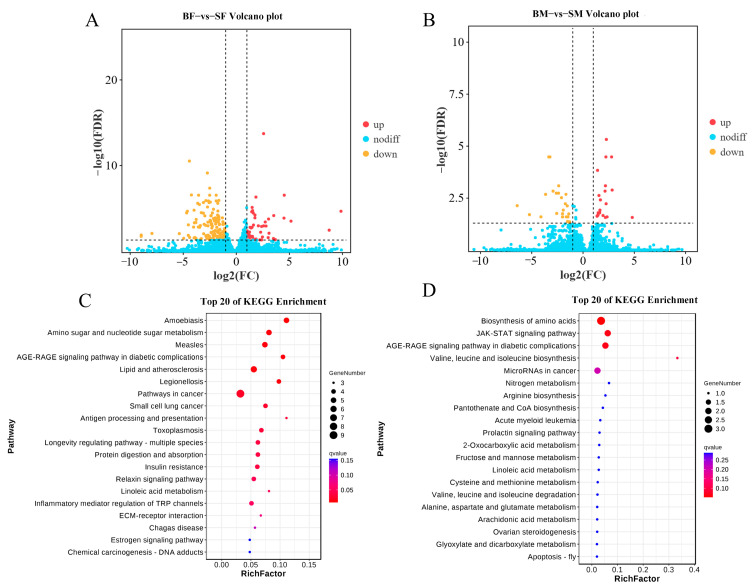
Transcriptome gene differential analysis and differential gene enrichment analysis. Volcano plot maps and summary of DEGs in the comparison of BFSF (**A**) and BMSM (**B**). The red and yellow dots show significantly upregulated genes and downregulated genes, respectively. Scatter plot of the top 20 KEGG enrichment pathways of DEGs in BFSF (**C**) and BMSM (**D**). The larger the enrichment factor in the scatter plot, the more significant the enrichment level of the DEGs in this pathway. The color of the dot represents the q value, and the size of the dot represents the number of DEGs in the pathway.

There were 208 different expression genes (DEGs) (49 upregulated and 159 downregulated) and 47 different expression genes (DEGs) (22 upregulated and 25 downregulated) identified in the BF-SF and BM-SM groups, respectively (Figure 1A,B). In the BF-SF group, 15 KEGG pathways were significantly enriched, including amino sugar and nucleotide sugar metabolism, the AGE-RAGE signaling pathway in diabetic complications and insulin resistance, and other pathways (Figure 1C). In the BM-SM group, DEGs were enriched mainly in 7 KEGG pathways, including the biosynthesis of amino acids, the JAK-STAT signaling pathway, the AGE-RAGE signaling pathway in diabetic complications, as well as valine, leucine, and isoleucine biosyntheses, MicroRNAs in cancer, nitrogen metabolism, and arginine biosynthesis (Figure 1D).

**Figure 2:**
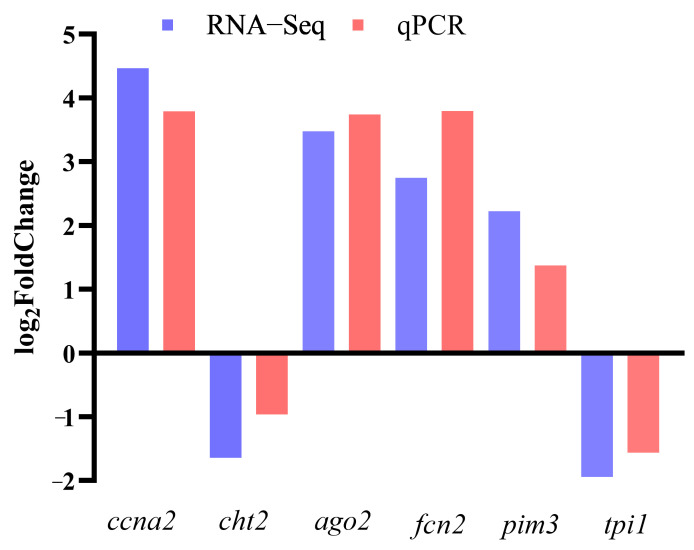
Comparison of relative gene expressions between RNASeq and qRTPCR data.

The expressions of six DEGs, *ccna2*, *cht2*, *ago2*, *fcn2*, *pim3*, and *tpi1*, were consistent with the results of the RNA-Seq analysis (Figure 2). These findings confirmed the integrity and reliability of the transcriptome analysis.

### 2.2. Differential Metabolites Analysis

**Figure 3:**
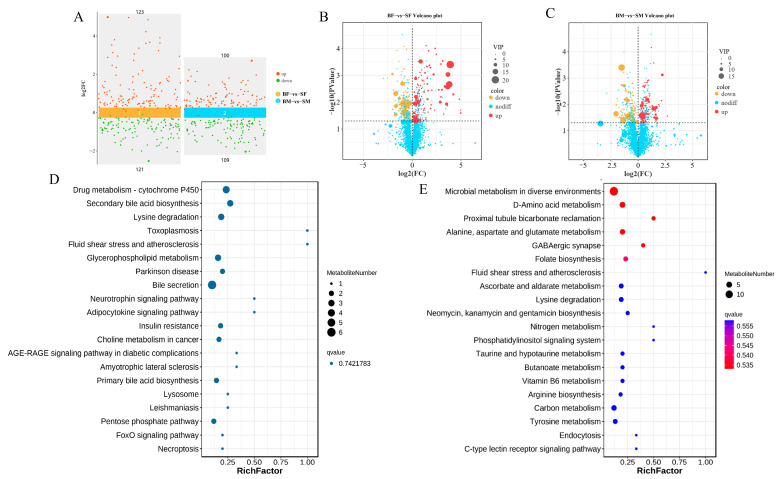
Differential metabolites analysis and differential metabolites enrichment analysis. (**A**) A scatter plot of multiple sets of differences between upregulated and downregulated DEMs. Volcano plot of the differential metabolites in BFSF (**B**) and BMSM (**C**) under anion (Pos) and cation (Neg) modes. Scatter plot of the top 20 KEGG enrichment pathways of DEMs in BFSF (**D**) and BMSM (**E**).

In total, 244 (123 upregulated and 121 downregulated) and 209 (100 upregulated and 109 downregulated) differential metabolites (DEMs) were identified from the comparisons of the BF-SF and BM-SM groups, respectively (Figure 3A–C). According to the result of KEGG enrichment, drug metabolism-cytochrome P450, secondary bile acid biosynthesis, and lysine degradation were the top three pathways in the BF-SF group (Figure 3D). Microbial metabolism in diverse environments, D-Amino acid metabolism, and proximal tubule bicarbonate reclamation were the top three pathways in the BM-SM group (Figure 3E).

### 2.3. Weighted Gene Co-Expression Network Analysis (WGCNA)

**Figure 4:**
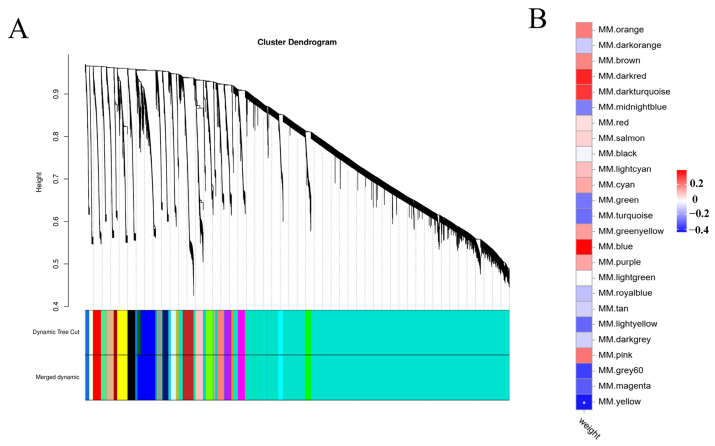
All gene expression results of WGCNA analysis. (**A**) The cluster tree shows 25 coexpressed gene modules identified by WGCNA with different colors. (**B**) Heat map showing module body weight correlations. The column corresponds to body weight. Each row corresponds to a module indicated by different colors. The red color indicates a positive correlation between the cluster and the tissue. The blue color indicates a negative correlation, * *p* < 0.05.

WGCNA was used to further elucidate the relationship between all genes and body weight. In total, 25 modules were identified, and the yellow module was closely related to body weight traits (Figure 4A). The yellow module, which contains 3097 genes, was negatively associated with body weight (Figure 4B).

**Figure 5:**
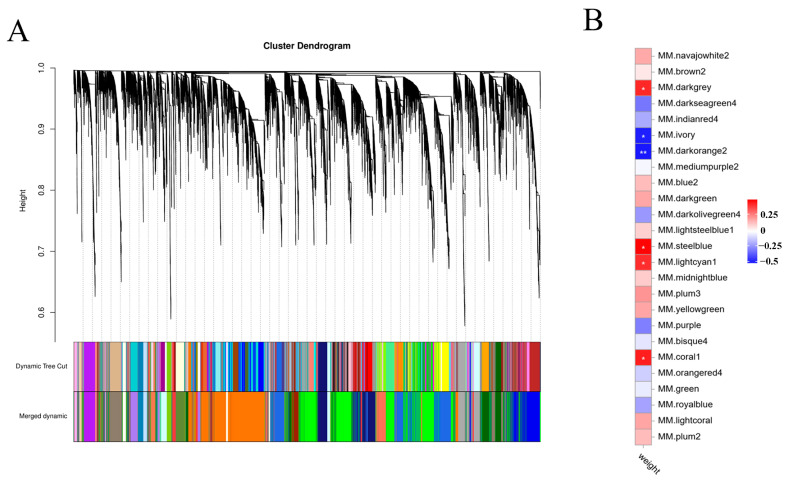
All metabolite results from the WGCNA analysis. (**A**) The cluster tree shows 25 metabolite modules identified by WGCNA with different colors. (**B**) Heat map showing module body weight correlations, * *p* < 0.05, ** *p* < 0.01.

WGCNA was used to elucidate the relationship between DEMs and body weight. In total, six significant modules were identified (Figure 5A,B). To further clarify key metabolites, the top 10 hub metabolites were screened from the six significant modules (Table 1).

### 2.4. Transcriptomics-Metabolomics Co-Regulated Network Analysis

**Figure 6:**
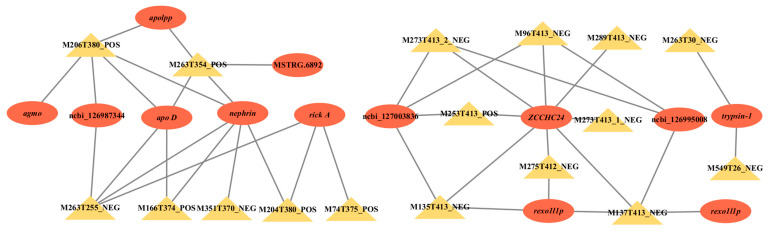
Co-expression network between key genes and hub metabolites related to the body weight of *E. sinensis*. The ellipses are key genes; rectangles are hub metabolites; gray lines indicate positive correlations.

**Figure 7:**
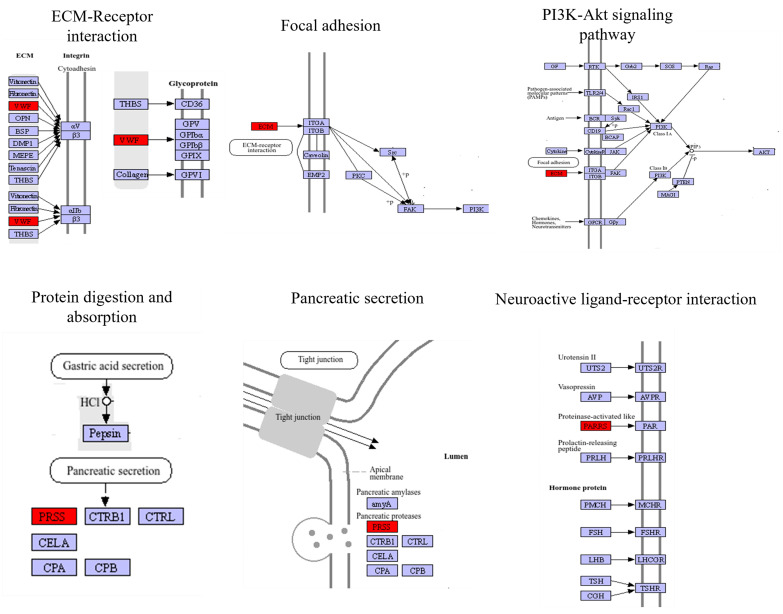
Key genes enrichment KEGG pathway.

The co-expression network diagram was established by screening the relationship pairs of the top 30 correlation coefficients (Figure 6). The key genes are apolipoprotein (*apolpp*), apolipoprotein D (*apo D*), alkylglycerol monooxygenase (*agmo*), nephrin, trypsin-1, arp2/3 complex-activating protein rick A (*rick A*), exonuclease GOR (*rexo1l1p*), putative exonuclease GOR (*rexo1l1p*), and zinc finger CCHC domain-containing protein 24 (*zcchc24*), and there were some uncharacterized genes (NCBI-126987344, NCBI-127003836, NCBI-126995008, MSTRG.6892). These key genes are mainly concentrated in six pathways, including the PI3K-Akt signaling pathway (ko04151), focal adhesion (ko04510), extracellular matrix (ECM)–receptor interaction (ko04512), neuroactive ligand–receptor interaction (ko04080), pancreatic secretion (ko04972), and protein digestion and absorption (ko04974) (Figure 7).

## 3. Discussion

Aquatic animals differ greatly in body size and growth strategies [21]. This is true even in the same growing environment [22,23]. In order to further explore the molecular mechanism of the growth differences in individuals, juvenile crabs cultured in the same pond were selected. After maturity, the crabs were classified by their body sizes, and the large and small body-sized crabs were separately sampled. To analyze the pattern of unsynchronized growth for large-body-size and small-body-size crabs, the muscles of crabs were sampled from each group for transcriptome and metabolome detection.

Based on the analysis of transcriptomic and metabolomic data, the transcriptome results showed that the amino sugar and nucleotide sugar metabolism and insulin signaling pathways were significantly enriched pathways in female crabs. Carbohydrate metabolism is crucial for the regulation of growth and development [24]. Amino sugar and nucleotide sugar metabolism regulate the absorption and assimilation of nutrients at lower levels [25]. The insulin-like peptide system in female mud crabs is involved in the regulation of yolk formation [26]. These signaling pathways in female crabs might be related to their reproductive needs, such as vitellogenesis. In male crabs, there are specifically and significantly enriched signaling pathways, including the biosynthesis of amino acids, the JAK-STAT signaling pathway, and nitrogen metabolism. The amino acid biosynthesis pathway is crucial for protein synthesis and muscle growth [27]. The enrichment of the JAK-STAT signaling pathway reflects differences in disease resistance or the stress response in crabs [28]. Nitrogen metabolism is associated with growth regulation-related metabolic adaptations [29]. The enhanced amino acid metabolism and nitrogen metabolism in male crabs might support their somatic growth, particularly muscle development. The results indicated that insulin resistance might constrain the growth of small body-sized female crabs, while large body-sized female crabs might prioritize energy storage through efficient glucose metabolism. For male crabs, large-body-sized crabs might achieve faster growth rates by enhancing amino acid biosynthesis and activating the JAK-STAT signaling pathway. The metabolite results showed that drug metabolism-cytochrome P450, secondary bile acid biosynthesis, and lysine degradation were the top three pathways in female crabs. In crustaceans, cytochrome P450 enzymes are involved in the synthesis of molting hormones (ecdysteroids) [30]. Cytochrome P450 2 plays an essential role in the mud crab *Scylla paramamosain* antioxidant defense and immune responses [31]. Bile acids play an important physiological role in modulating a number of hepatic and intestinal functions and exerting systemic effects such as increasing energy expenditure and improving insulin sensitivity [32]. Lysine improves the growth performance, molting frequency, and lipid metabolism of postlarval mud crab *Scylla paramamosain* [33]. The enriched pathways in female crabs indicated their higher demand for lipid metabolism, particularly for energy storage, including vitellogenesis during ovarian development. In male crabs, the three pathways highlighted the significance of microbial interactions and acid-based balance regulation. These results showed that different body sizes of female crabs primarily exhibit lipid metabolism and stress-response regulatory pathways, while different body sizes of male crabs mainly showed pathways related to microbial community maintenance and osmoregulation.

Based on the integrated analysis of transcriptomic and metabolomic data, a total of 13 key genes have been identified. Among these, NCBI-126984229 (*apolpp*) is enriched in the following pathways: PI3K-Akt signaling pathway, ECM–receptor interaction, and focal adhesion. NCBI-127006026 (*trypsin-1*) is enriched in the following pathways: Neuroactive ligand–receptor interaction, pancreatic secretion, and protein digestion and absorption. NCBI-127007012 (*agmo*) encodes an enzyme that catalyzes the breakdown of alkylglycerols and is involved in processes such as cell membrane composition, embryonic development, neural development, and metabolic regulation [34]. NCBI-127009577 (*rick A*) is a highly elastic protein commonly found in the exoskeletons of arthropods. During insect development, the expression of the resilin gene influences their elasticity and locomotor capabilities. The elasticity of insects aids in flight and movement, thereby impacting their survival and reproduction [35]. NCBI-127004947 (*nephrin*) encodes the NPHS protein, which is involved in the regulation of kidney function [36]. NCBI-127002774 (*rexo1l1p*), NCBI-127002775 (*rexo1l1p*), and NCBI-126982641 (*zcchc24*) are involved in RNA metabolism or the regulation of gene expression [37]. Therefore, several genes were studied and analyzed in this study.

*Apolpp* is a member of the *apoB* family, conserved across the animal kingdom [38]. *Apolpp* is the *apoB* homologous apolipoprotein in fruit flies [38]. The *apolpp* is involved in lipid transport and energy metabolism [39]. A previous study had shown that inhibiting *apoLpp* in the fat body significantly reduces systemic lipid levels in fruit flies fed a standard diet, highlighting the critical contribution of the fat body *apoLpp* to systemic lipid metabolism [40]. In this study, *apolpp* is involved in the ECM–receptor interaction, focal adhesion, and PI3K-Akt signaling pathways. As shown in the pathway diagram, when receptors such as integrins bind to the ECM, they recruit various signaling molecules and cytoskeletal proteins to form the focal adhesion complex. Focal adhesion can indirectly regulate the PI3K-Akt pathway by activating downstream signaling molecules such as FAK. In the large body-sized crab, the *apolpp* is upregulated in the three pathways. In the ECM–receptor interaction, the ECM is composed of glycoproteins, collagen, and proteoglycans, forming a complex, dynamic structure. In the process of myogenesis, integrins participate in the interaction between cells and the ECM, as well as the formation of sarcomeres, which play a significant role in the formation and growth of muscles [41]. In addition, according to the relevant report, the integrin genes in fast-growing oysters are upregulated in the ECM–receptor interaction signaling pathway, indicating active interactions between cells and the extracellular matrix (ECM) [42]. Focal adhesions are dynamic connecting structures between cells and the extracellular matrix (ECM), primarily mediated by receptors such as integrins. They play a crucial role in regulating cell adhesion, migration, proliferation, differentiation, and signal transduction [43]. Focal adhesions activate downstream signaling pathways, such as the FAK, Src, PI3K-Akt, and MAPK pathways, through integrins, thereby regulating cell growth, survival, and differentiation [44]. The PI3K-Akt signaling pathway plays a crucial role in the growth and development of animals. It is a highly conserved signaling pathway that influences embryonic development, tissue formation, and organ function by regulating cell growth, proliferation, survival, metabolism, and differentiation. During animal growth and development, the homeostasis of glucose metabolism is crucial for maintaining animal health. The PI3K-Akt signaling pathway sustains cellular energy balance by regulating glucose uptake, glycogen synthesis, and lipid metabolism [45]. Han [46] found that gene variations in the PI3K-Akt signaling pathway can affect the growth performance of animals. In addition, in this study, the transcriptome results showed that the ECM–receptor interaction was the main enrichment pathway of differential genes in female crabs. Differential genes of large-body-sized crabs enriched in this pathway are also upregulated. Therefore, key biological processes such as *apoIpp*, as well as the ECM–receptor interaction, cell adhesion, and PI3K-Akt signaling pathway, play a central role in growth regulation.

*Trypsin-1* activates the conversion of trypsinogen to trypsin, thereby initiating the digestive enzyme cascade reaction and promoting the digestion and absorption of proteins [47]. *Trypsin-1* influences the absorption and utilization of nutrients by regulating protein digestion, thereby indirectly affecting growth and development. Mutations in *Trypsin-1* can lead to enterokinase deficiency, a malabsorption disorder characterized by diarrhea and growth retardation [47,48]. The normal development of the intestinal tract is crucial for the overall growth of an individual. Similarly, in our study, we also found that *trypsin-1* is involved in the pancreatic secretion protein digestion and absorption pathway. *Trypsin-1* in large-body-sized crabs is upregulated. Currently, *Trypsin-1* is primarily recognized for its association with digestive functions, influencing animal growth by regulating the digestion and absorption of nutrients as well as intestinal development. In addition, in our study, *Trypsin-1*, involved in the neuroactive ligand–receptor interaction, was also upregulated. This result indicates that *trypsin-1* in large-body-sized crabs exhibits a high digestive capacity and plays a crucial role in neural development.

In this study, we found several other genes, such as *rick A*, *agmo*, *nephrin*, *rexo1l1p*, and *zcchc24. Rick A* induced actin polymerization [49], while the Arp2/3 complex alone is a weak actin nucleator, the rate of Arp2/3-mediated actin polymerization is increased in the presence of a class of proteins known as nucleation-promoting factors [50,51,52,53]. Meanwhile, AMGO is the only known alkylglycerol monooxygenase, an orphan tetrahydrobiopterin-dependent enzyme that cleaves the ether linkage in alkylglycerols. AMGO is capable of catalyzing the breakdown of alkylglycerols, producing glycerol and corresponding aldehydes [54]. AMGO is involved in the metabolism of ether lipids, which play significant roles in cell signaling and membrane structure [34]. By regulating ether lipid metabolism, AMGO may influence cell growth, differentiation, and signaling. Mutations, copy number variations, and deletions in the *agmo* gene can lead to microcephaly and neurodevelopmental disorders [55,56]. The relationship between NERPHRIN and animal growth and development is mainly reflected in kidney development and function. The *nephrin* gene encodes a transmembrane protein known as NERPHRIN, which is primarily expressed in glomerular podocytes [36]. The absence or mutation of the NERPHRIN can lead to abnormal glomerular development, thereby affecting kidney function. In humans, mutations in the *nephrin* gene can cause Congenital Nephrotic Syndrome (CNS), which is characterized by growth retardation [57]. REXO1L1P and ZCCHC24 are involved in RNA metabolism or the regulation of gene expression [37,58]. Thus, proteins regulated by these genes influence animal growth and development by participating in tissue specificity, lipid metabolism, kidney development, RNA metabolism, and gene expression pathways.

## 4. Materials and Methods

### 4.1. Ethics Statement

This study was conducted in accordance with the guidelines of the Animal Care and Use Committee of Nanjing Agricultural University, Nanjing, China. The protocol was approved by the Animal Research Committee of Nanjing Agricultural University (permit number: SYXK (XU) 2023–0108; approved on 6 January 2023).

### 4.2. Experimental Design and Sample Collection

The cultivation of *E. sinensis* was conducted at the Yangzhong Experimental Base (Zhenjiang, China), Freshwater Fisheries Research Center, Chinese Academy of Fishery Sciences, from January to November 2023. The cultivation conditions and management methods for *E. sinensis* are described in a previous study [59]. A total of 72 healthy crabs (18-month-old mature) cultivated in the same pond were divided into 4 groups, with 18 crabs in each group, namely large-sized female crabs (BF, 196.42 ± 3.12 g), large-sized male crabs (BM, 248.01 ± 3.89 g), small-sized female crabs (SF, 111.06 ± 2.51 g) and small-sized male crabs (SM, 160.50 ± 3.42 g). They were anesthetized on ice before sampling to measure the wet weight (using an electronic scale, accurate to 0.01 g) and carapace length and width (using a digital caliper, accurate to 0.01 mm). Then, the muscle tissue of the BF, BM, SF, and SM groups was collected for transcriptome and metabolomics analyses. All samples were immediately frozen in liquid nitrogen and then stored at −80 °C before use.

### 4.3. Transcriptome Sequencing and Differential Expressed Genes Analysis

We constructed 24 sequencing libraries, six libraries for each group. The RNA for the muscle tissue was extracted by using a Trizol reagent kit (Invitrogen, Carlsbad, CA, USA). Furthermore, cDNA library construction and sequencing were performed on an Illumina NovaSeq 6000 platform, as previously described [59,60]. The quality check of raw sequence reads was performed via FastQC v0.11.2 [61]. Transcriptome assembly was accomplished using Trinity [62]. Gene function was annotated based on the following databases: Nr, the UniProt-SwissProt, KEGG, KOG, COG, GO, and the Pfam database. Gene expression levels were estimated via RSEM [63] for each sample. Gene levels were estimated as fragments per kilobase of transcript per million mapped reads (FPKM). Differential gene expression between the nitrite-treated and control groups was determined using DESeq2 (version 1.26.0). An absolute log2 fold change (FC) > 1 and false discovery rate (FDR) < 0.05 were set as the thresholds for differentially expressed genes (DEGs). The DEGs were further subjected to the GO and KEGG enrichment analyses [64].

### 4.4. qRT-PCR Validation

To validate the reliability of the transcriptome data, six DEGs (*ccna2*, *cht2*, *ago2*, *fcn2*, *pim3*, and *tpi1*) were selected to measure their mRNA expression levels by qRT-PCR. Total RNA from each group of 18 muscles was extracted with RNAiso Plus reagent (Takara Bio Inc., Beijing, China) and removed from contaminating genomic DNA with RNase-free DNase (Takara Bio Inc., Beijing, China). The OD values at 260 and 280 nm and their ratios (A260/A280 were 1.8–2.1) were used to assess the quality and quantity of the extracted RNA. cDNA was synthesized using 1 μg of extracted RNA and used to detect the expression of the target genes by quantitative real-time PCR (qRT-PCR) using the TB Green™ Premix Ex Taq™ II kit (Takara Bio Inc., Beijing, China). Furthermore, qRT-PCR was performed as follows: 95 °C for 30 s, followed by 40 cycles of 95 °C for 5 s and 60 °C for 1 min. The 2^−∆∆Ct^ method [65] was used to calculate the relative expression levels of the target genes with *β-actin* as the housekeeping gene. The primers are shown in Table 2.

### 4.5. Metabolite Extraction and LC-MS/MS Analysis

The metabolomics analysis based on LC-MS/MS was performed on 24 muscle samples in *E. sinensis* with six bio-replicates of each group. Tissues of 80 mg were cut on dry ice and homogenized with 200 μL of H_2_O and five ceramic beads. Then, 800 μL of methanol/acetonitrile (1:1, *v*/*v*) was added to the homogenized solution for metabolite extraction. After the mixture was centrifuged for 15 min (14,000× *g*, 4 °C), the supernatant was dried and re-dissolved in 100 μL of acetonitrile/water (1:1, *v*/*v*) solvent. LC-MS/MS analysis was performed using a UHPLC system (Agilent 1290 Infinity LC, Thermo Fisher Scientific, Waltham, MA, USA) and an AB TripleTOF 6600 mass spectrometer was used for the acquisition of primary and secondary spectra of the samples.

Data were normalized to exclude QC sample variables with a relative standard deviation (RSD) greater than 30%. Autoscaling, mean-centering, and scaled-to-unit variance (UV) were performed before conducting Orthogonal Projections to Latent Structures Discriminant Analysis (OPLS-DA) [66] to perform automatic modeling analysis. Then, DEMs were identified by Variable Importance in the Projection, VIP > 1 and *p* < 0.05 [67], and analyzed with KOBAS and Fisher’s exact tests to explore the enriched KEGG pathways [68].

### 4.6. Transcriptomics-Metabolomics Co-Regulated Network Analysis

We first applied Weighted Gene Co-expression Network Analysis (WGCNA) to identify the modules significantly correlated with traits in both the transcriptomic and metabolomic datasets. Subsequently, the top 10 metabolites from the trait-associated modules were screened using Cytoscape v3.7.2. Finally, correlation analysis was performed between the trait-related genes and these selected metabolites. Pearson correlations between DEGs and DEMs were calculated using the Psych package from the R project (V3.6.2). Further screening results showed that the correlation coefficient was ≥0.80 with a *p*-value < 0.05. All DEGs and DEMs were mapped to the KEGG pathway database to screen for the primary genes and signaling pathways [69]. Cytoscape software (V3.7.2) was used to map the network regulation of the related results.

## 5. Conclusions

By integrating transcriptomic and metabolomic data, this study is the first to systematically reveal the differences in molecular regulatory mechanisms among crabs of different body sizes. This study found that larger female crabs exhibit significantly enhanced digestive functions, primarily reflected in the upregulation of *trypsin-1* expression. This gene plays a pivotal role in pancreatic secretion, protein breakdown, and neuroactive ligand–receptor interactions, thereby regulating the growth and development of crabs. Further, a variety of tissue-specific proteins, such as APOLPP, RICK A, AGMO, and NEPHRIN, as well as REXO1L1P and ZCCHC24, which are related to RNA metabolism and gene expression stability, indirectly influence the growth and development of the crab through their respective functional pathways. In addition, the results revealed that key KEGG pathways, such as the ECM–receptor interaction, cell adhesion, and PI3K-Akt signaling pathway, play a central role in growth regulation.

## Figures and Tables

**Table 1:** Top 10 hub metabolites in each module.

Modules					
Darkgrey	Ivory	Darkorange2	Steelblue	Lightcyan1	Coral1
M934T411_2_POS	M273T413_1_NEG	M1143T298_POS	M74T375_POS	M263T30_NEG	M368T388_NEG
M1173T410_POS	M96T413_NEG	M499T298_NEG	M166T374_POS	M527T31_NEG	M513T382_NEG
M1162T410_4_POS	M724T155_POS	M376T298_POS	M206T380_POS	M237T31_NEG	M552T387_NEG
M1172T410_4_POS	M275T412_NEG	M822T298_POS	M75T374_POS	M551T30_NEG	M697T382_NEG
M1172T410_1_POS	M289T413_NEG	M374T298_NEG	M351T370_NEG	M309T28_POS	M144T306_NEG
M1162T410_1_POS	M135T413_NEG	M251T298_POS	M506T394_NEG	M573T26_1_NEG	M475T377_NEG
M1162T410_3_POS	M253T413_POS	M626T298_POS	M204T380_POS	M249T30_NEG	M551T383_NEG
M1181T410_2_POS	M439T153_1_POS	M624T298_NEG	M263T255_NEG	M262T55_NEG	M130T295_POS
M1172T410_2_POS	M273T413_2_NEG	M874T298_NEG	M242T404_1_POS	M549T26_NEG	M446T389_POS
M1182T410_1_POS	M137T413_NEG	M564T298_POS	M263T354_POS	M187T86_POS	M611T383_NEG

**Table 2:** The primers and sequences referred to in the experiment.

Primers	Position	Primer Sequence	Accession No.
*ccna2*	Forward	CACCCATATGGTCAAGGAGCTAA	XM_050835160.1
	Reverse	CTCTGATATGGAGAACTCCAGGC	
*cht2*	Forward	AACACCACCTACACCATGAAGTC	XM_050884678.1
	Reverse	TTCTTGTCCACGAACCTCTCAAG	
*ago2*	Forward	GATGGAGTAGGAAAGTCAGGCAA	XM_050835160.1 P
	Reverse	TGATTGTGTTGTCCACTGTTGTG	
*fcn2*	Forward	TGAATCACTACGACAACCGACC	XM_050849112.1
	Reverse	GTACACTTGGCGAATGCCCTTG	
*pim3*	Forward	CGACTTAATGCTACAGGTGGTGA	XM_050869002.1
	Reverse	CAGGTTCTCGTCCTTGATGTCG	
*tpi1*	Forward	ACAGAACTGCTACAAGGAACCAC	XM_050875155.1
	Reverse	CCAAGGATCACCCACTCACATC	
*β-actin*	Forward	TCATCACCATCGGCAATGA	XM_050843215.1
	Reverse	TTGTAAGTGGTCTCGTGGATG	

## Data Availability

All the data of the transcriptome are available at the NCBI SRA database (PRJNA1240068).

## Supplementary Materials

The following supporting information can be downloaded at: https://www.ncbi.nlm.nih.gov/bioproject/PRJNA1240068, accessed on 10 May 2025.
